# Image quality, diagnostic accuracy, and potential for radiation dose reduction in thoracoabdominal CT, using Sinogram Affirmed Iterative Reconstruction (SAFIRE) technique in a longitudinal study

**DOI:** 10.1371/journal.pone.0180302

**Published:** 2017-07-05

**Authors:** Michael Scharf, Stephanie Brendel, Katja Melzer, Christian Hentschke, Matthias May, Michael Uder, Michael M. Lell

**Affiliations:** 1 Department of Radiology, Friedrich-Alexander-University Erlangen-Nuremberg, Erlangen, Germany; 2 Institute of Sport Science and Sport, Friedrich-Alexander-University Erlangen-Nuremberg, Erlangen, Germany; Maastricht University Medical Centre, NETHERLANDS

## Abstract

**Objective:**

To step-wise evaluate image quality of sinogram-affirmed iterative reconstruction (SAFIRE) in reduced-dose (RD) thoracoabdominal computed tomography (CT) compared to full-dose (FD) and RD filtered back projection (FBP) in a longitudinal study.

**Materials and methods:**

122 patients were included in this prospective study. 49 patients (14 men: mean age ± SD, 56±0.4 years; 35 women: 58±1.3 years) completed FD, RD1 (80%-dose) and RD2 (60%-dose) thoracoabdominal CT. Each CT dataset was reconstructed with FBP and SAFIRE. For quantitative image analysis image noise was measured in defined tissue regions. Qualitative image evaluation was performed according to the European Guidelines on Quality criteria for CT. Additionally artifacts, lesion conspicuity, and edge sharpness were assessed.

**Results:**

Compared to FD-FBP noise in soft tissue increased by 12% in RD1-FBP and 27% in RD2-FBP reconstructions, whereas SAFIRE lead to a decrease of 28% (RD1) and 17% (RD2), respectively (all p <0.001). Visually sharp reproduction, lesion conspicuity, edge sharpness of pathologic findings, and overall image quality did not differ statistically significant between FD-FBP and RD-SAFIRE datasets. Image quality decreased in RD1- and RD2-FBP compared to FD-FBP, reaching statistically significance in RD2 datasets (p <0.001). In RD1- and RD2-FBP (p <0.001) streak artifacts were noted.

**Conclusion:**

Using SAFIRE the reference mAs in thoracoabdominal CT can be reduced by at least 30% in clinical routine without loss of image quality or diagnostic information.

## Introduction

The increase in radiation exposure from diagnostic testing is of growing concern. From 1980 to 2010 the annual per capita diagnostic radiation dose in the United States increased four to five times from 0.5 mSv to 2.3 mSv [1, 2]. Although CT accounts for less than 20% of all radiological examinations performed, it is responsible for more than two thirds of the cumulative effective dose in medical imaging [3]. Especially in patients with cancer who undergo frequent follow-up CT examinations collective radiation burden is high.

To lower radiation exposure, different image acquisition techniques like tube current modulation [4], automatic exposure control (AEC) [5], and tube potential selection [6] have been developed. Tube current correlates to dose in a linear fashion and hence, reductions of the tube current lead to a decline in radiation dose values. The challenge in CT is to keep radiation dose to a minimum, while guaranteeing diagnostic image quality. Decreasing radiation exposure is associated with an increase in image noise and at a certain level results in unacceptable loss of diagnostic performance [7]. To overcome these limitations and improve image quality in reduced-dose (RD) CT, iterative reconstruction techniques, as a rather old method for image optimization [8], are increasingly applied in clinical routine. Each CT vendor has introduced a different iterative reconstruction technique [9]. Sinogram-affirmed iterative reconstruction (SAFIRE, Siemens Healthcare, Forchheim, Germany) is an algorithm applied on Siemens CT systems without Stellar detectors. It has been demonstrated in previous studies, that the higher image noise of RD abdominal CT can be minimized with iterative reconstruction algorithms, thus enabling substantial radiation dose savings with preserved diagnostic image quality [10, 11]. For the SAFIRE-algorithm up to 75% dose reduction have been reported in abdominal CT [12, 13]. Previous studies employing SAFIRE were predominantly performed with dual-source CT scanners. Mostly a fixed splitting of tube current to both X-ray tubes (e.g., 50% of total reference mAs each) for the reconstruction of half-dose and full-dose (FD) images was used [7, 14–16]. Few investigations acquired different radiation exposure levels (e.g., 100%, 75%, 50%, 37.5%, 25%, and 12.5%) from the same CT acquisition [12]. The purpose of this prospective longitudinal investigation is to evaluate the effects of SAFIRE on objective and subjective image quality in comparison to FBP in a clinical setting on a single source CT system. Therefore, three consecutive thoracoabdominal CT scans with a step-wise dose reduction from 100% to 80% and 60% were performed.

## Materials and methods

### Study population

The institutional review board of the Faculty of Medicine of the University of Erlangen-Nuremberg approved the study, and written informed consent was obtained from all subjects. The investigation was conducted according to the principles of the Helsinki Declaration. Patients were recruited and examined between May 2013 and April 2015. Exclusion criteria from study were history of allergic reaction to iodined contrast material, renal insufficiency (glomerular filtration rate < 45 mL/min/1.73m^2^) or hyperthyroidism. Significant changes in clinical performance (change of body weight > 4 kg), change of lesion size > 20%, appearance/disappearance of ascites) between the three examinations were further exclusion criteria.

One hundred and twenty-two consecutive patients, referred for tumour staging, underwent FD thoracoabdominal CT. There were several drop-outs due to death (n = 14), exclusion criteria from study (change in lesion size or body weight, n = 28), and missing or restaging at a different institution within the study period (n = 31). Finally, data of FD scans and two RD follow-up CT examinations with 20% and 40% dose reduction were available in forty-nine patients (14 men: mean age ± SD, 56 ± 10.4 years; 35 women: 58 ± 11.3 years). Underlying tumour disease was as follows: breast cancer, n = 17; ovarial/cervical carcinoma, n = 16; colon cancer, n = 6; renal tumour, n = 4; others, n = 6).

Patients’ characteristics are provided in Table 1. One patient was underweight (body mass index, BMI < 18.5 kg/m^2^), 28 were of normal weight (BMI = 18.5–24.9 kg/m^2^), 13 patients were overweight (BMI = 25.0–29.9 kg/m^2^), and seven patients were obese (BMI > 30.0 kg/m^2^).

**Table 1:** Patients’ physical characteristics.

*Physical characteristics*	men (n = 14)	women (n = 35)
Age (yr)	56±10.4	58±11.3
Height (m)	1.75±0.07	1.63±0.06
Body weight (kg)	75.3±13.2	67.7±12.6
BMI (kg/m^2^)	27.3±5.1	24.5±4.2

Data are indicated as mean ± standard deviation.

BMI = body mass index.

### CT protocol

CT examinations were performed on a 128-slice multidetector-row CT scanner (Somatom Definition AS+, Siemens Healthcare, Forchheim, Germany). All CT data was acquired with activated automatic exposure control (CARE Dose 4D, Siemens Healthcare, Forchheim, Germany). CT scan parameters for FD and RD examinations were: tube voltage, 120 kV; slice collimation, 128 x 0.6; rotation time, 0.5 seconds; pitch, 0.9. Tube current time product was 210 reference mAs in FD, 170 ref. mAs in RD1 (80% dose), and 130 ref. mAs in RD2 (60% dose) CT, respectively. 100 ml of Iopromide (Ultravist 370, Bayer-Schering Healthcare, Berlin, Germany) was injected through a 20-gauge antecubital vein catheter with a flow rate of 3 mL/s, followed by 50 mL 0.9% saline. Scan delay was set at 70 seconds after the start of contrast injection to achieve a portal venous phase. A craniocaudal scan direction was chosen and all CT imaging data were acquired with a breath hold in deep inspiration to eliminate respiratory motion artifacts.

### Image reconstruction

All examinations were reconstructed with FBP and SAFIRE with a reconstruction field of view adapted to body habitus. Thick slices (slice thickness / increment, 5 mm / 5 mm) were used for image quality and quantity assessment, thin slices (0.75 mm / 0.5 mm) for 3D reconstructions. A standard soft tissue convolution kernel (B31) was applied for FBP datasets.

Five presets (strength 1–5) are available for noise suppression with SAFIRE. We used a medium strength level of 3 in all patients.

### Image analysis

**Fig 1:**
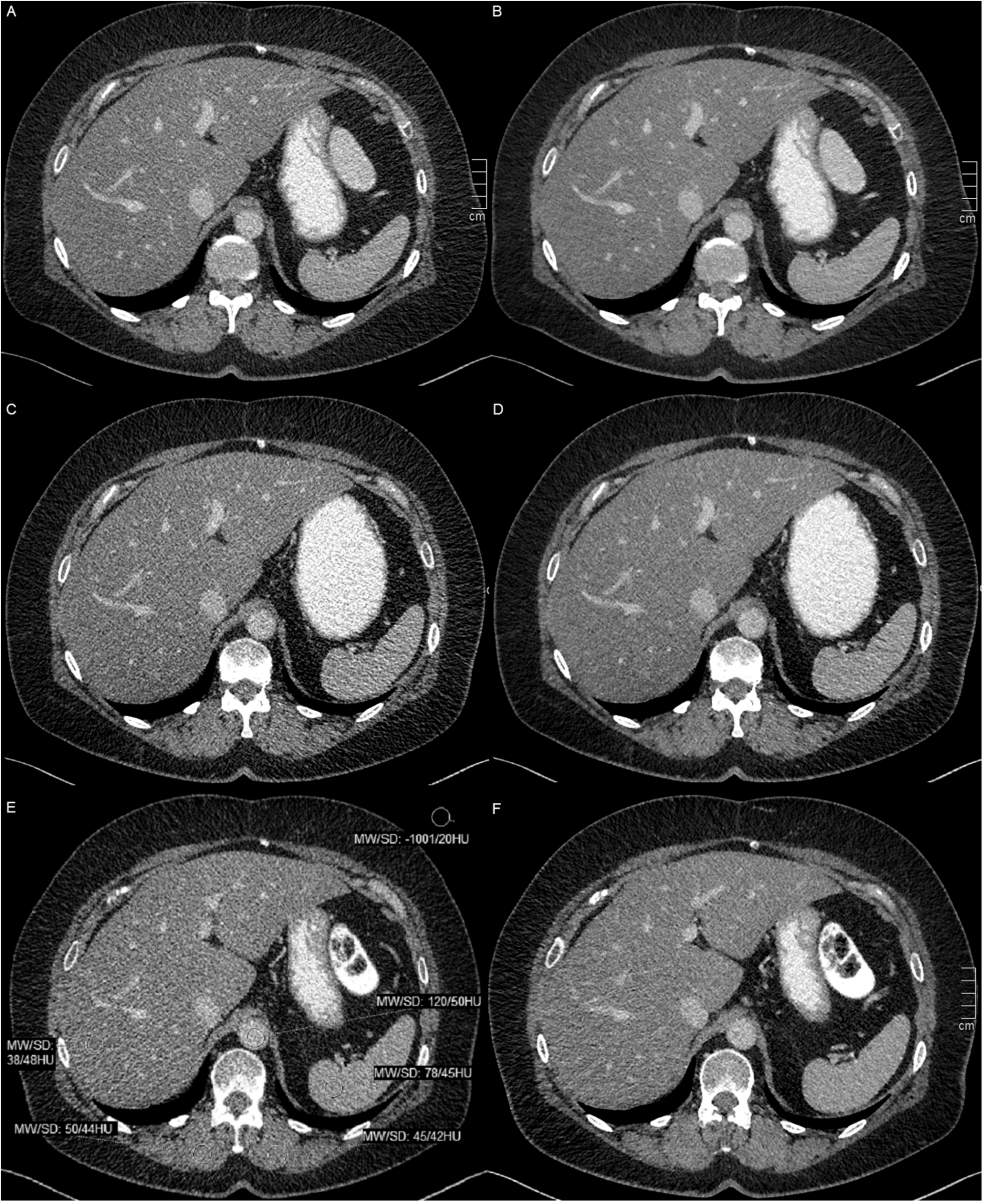
Quantitative image assessment. Quantitative image analysis in a 65-year-old woman with body mass index of 27. Transverse images obtained with FD-FBP (A), FD-SAFIRE (B), RD1-FBP (C), RD1-SAFIRE (D), RD2-FBP (E), and RD2-SAFIRE (F). FD = full dose, RD = reduced dose (RD1 = 80% dose, RD2 = 60% dose), FBP = filtered back projection, SAFIRE = sinogram-affirmed iterative reconstruction. Image noise was measured in six defined soft tissue regions (liver, spleen, aorta, gallbladder, left and right erector spinae muscles) and air. For reasons of clarity regions of interest are only shown in RD2-FBP (E). Compared to FD-FBP mean image noise in soft tissue significantly (p <0.001) increased in RD1- and RD2-FBP reconstructions whereas mean image noise in RD-SAFIRE reconstructions significantly decreased (p <0.001).

Datasets were transferred to a 3D image processing workstation (Syngo Via, Siemens Healthcare, Forchheim, Germany) after removing patient and scanning information and the type of image reconstruction. To assess subjective and objective image quality, CT datasets were independently analyzed in random order by two board certified radiologists with 5 and 19 years of experience in thoracoabdominal CT imaging, respectively. At the time of study conduction readers experience with SAFIRE was one and three years, respectively. Radiologist applied iterative reconstruction algorithms (e.g., Iterative Reconstruction in Image Space, IRIS) for three and ten years before this study, respectively. All six image datasets of each subject were simultaneously displayed with a preset soft tissue window (W/C = 380/50 HU). Brightness and resolution on the viewing monitor was identical during the reading sessions. For objective image quality image noise in the datasets was measured as the standard deviation of the pixel values from a homogeneous, circular region of interest (ROI) in soft tissue (liver segment 6, spleen, gallbladder, aorta, left and right erector spinae muscles, Fig 1) and air. Size of ROI in soft tissue and air was 1.5 cm^2^. Due to prior surgery the gallbladder (n = 8) or spleen (n = 3) was not present in every patient. In the liver and spleen, care was taken not to include any major blood vessel or lesion in the ROI. Using a copy function, the identical ROI location was used for all datasets of every single subject.

Subjective image quality was assessed according to the European Guidelines on Quality Criteria for CT [17]. Visually sharp reproduction of anatomic regions (liver parenchyma and common biliary tract, pancreas, kidney, vessels, lymph nodes and adipose tissue) was rated on a dichotomic scale (1, yes; 2, no). Image noise and spatial resolution were evaluated on a 3 point scale (1, too little; 2, optimum; 3, too much) and overall diagnostic acceptability on a 4 point scale (1, fully acceptable; 2, probably acceptable; 3, only acceptable under limited conditions; 4, unacceptable). In addition, each radiologist assessed for presence of any image artifacts which were categorized into windmill or helical artifacts, streak or beam hardening artifacts, and truncation artifacts. Anatomic location of each artifact was recorded. Finally, the influence of artifacts on diagnostic evaluation was graded on a four point scale (1 = no artifact, 2 = minor artifacts not affecting the visualization of any structure, 3 = artifacts affecting visualization of normal structure, and 4 = major artifacts affecting diagnostic information).

Pathologic findings were assessed for lesion conspicuity and edge sharpness. Lesion conspicuity was divided in five categories (0, no lesion; 1, fully visible; 2, predominantly visible; 3, visible under limited conditions; 4, not visible). Edge sharpness was graded on a four point scale (1, totally sharp; 2, predominantly sharp; 3, sharp with limitations; 4, not sharp).

### Radiation dose estimates

Radiation dose parameters were assessed from the patient protocol. The average effective dose (ED) was retrospectively calculated by multiplying the dose-length product (DLP) value by a region-specific conversion factor (κ = 0.015 mSv x mGy^-1^ x cm^-1^) [18].

### Statistical analysis

Computations were performed using SPSS version 21.0 (SPSS, Chicago, Illinois, USA). All variables are expressed as mean value ± standard deviation. Throughout the analysis, a 2-sided p value <0.05 was considered statistically significant. To compare the density values and image noise within the reconstructed datasets 1-way ANOVA (analysis of variance) and subsequent Bonferroni post hoc tests were performed. We conducted qualitative image analysis with nonparametric Friedman-ANOVA and subsequent post hoc tests as proposed by Conover [19]. Interobserver agreement on image quality was evaluated using Cohen's kappa statistics.

## Results

All CT scans were successfully completed and considered satisfactory with regard to diagnostic image quality. There was no significant difference in the BMI of the patients included in the final analysis between the three CT scans: mean BMI ± standard deviation was 26.2 ± 5.7 kg/m^2^ for the initial scan, 25.9 ± 5.3 kg/m^2^, and 25.7 ± 5.4 kg/m^2^ for the two follow-up scans. Scan range length was 60.8 ± 14.2 cm (FD), 62.1 ± 15.4 cm (RD1), and 59.9 ± 16.2 cm (RD2). Mean scan length was 61 ± 12 cm, resulting in a mean scan time of 10 ± 3 seconds. Image reconstruction of 0.75 mm / 0.5 mm datasets with FBP was performed in a mean time of 38 ± 5 seconds. The SAFIRE-algorithm took 49 ± 8 seconds. Thus, SAFIRE was associated with a 1.3–fold increase in reconstruction time.

### Radiation dose estimates

**Fig 2:**
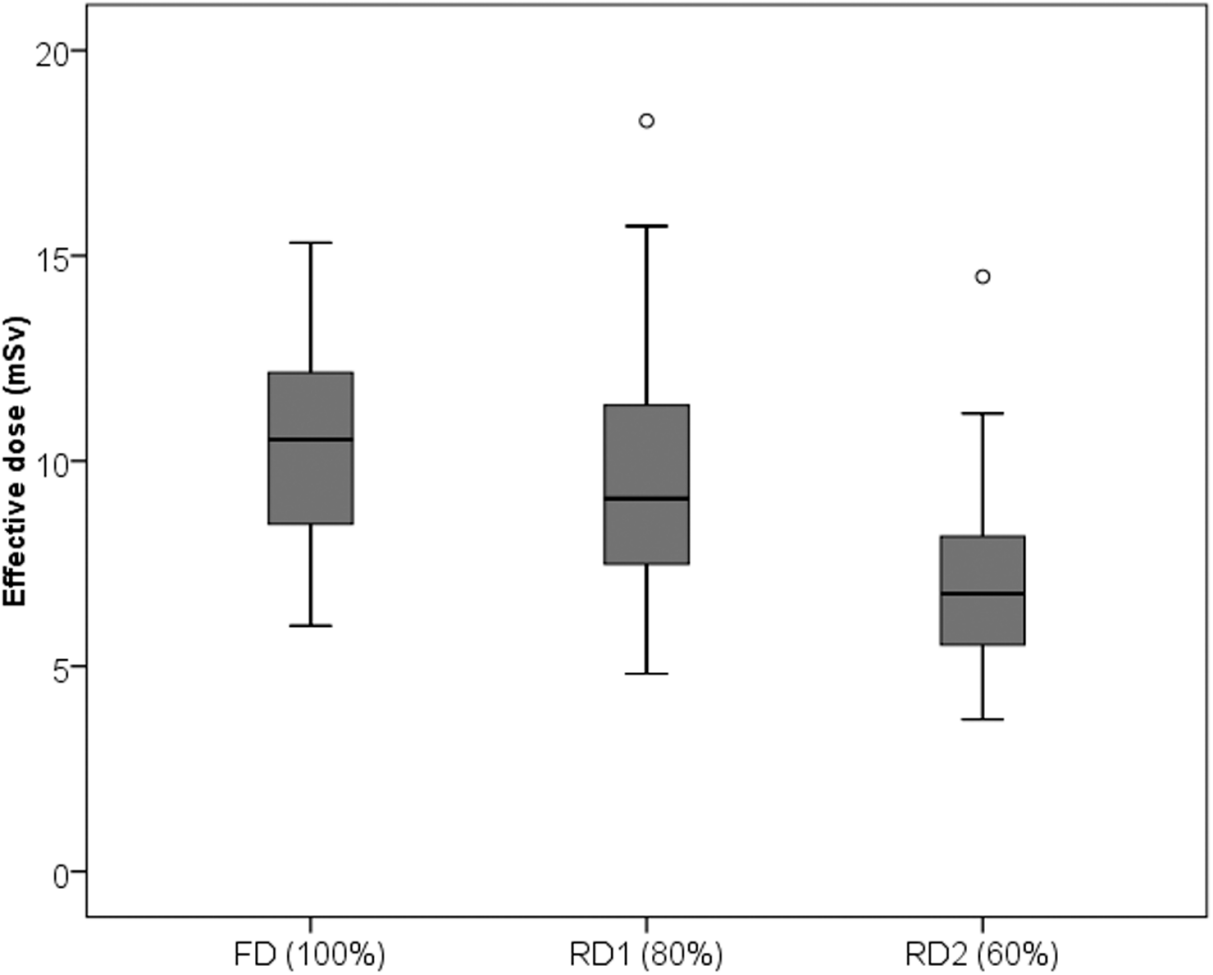
Potential of radiation dose savings in reduced-dose thoracoabdominal CT. Box and whisker plot demonstrates potential for significant radiation dose reduction in reduced-dose (RD1 = 80% dose; RD2 = 60% dose) thoracoabdominal CT using SAFIRE compared to full-dose (FD) scans. ED = effective dose (mSv). Both outliers above average study group dose values in RD1- and RD2-CT scans were obese patients.

Parameters of radiation dose are detailed in Table 2. Although the reference mAs in the two RD scans was reduced in 20% steps, automatic exposure control lead to a decrease in volume CT dose index (CTDI_vol_) by 6.7% and 30.5%, respectively. Box and whisker plot demonstrates ED reduction in RD compared to FD scans (Fig 2). Both outliers above average study group dose values in RD1 and RD2 were obese patients.

**Table 2:** Radiation dose estimates.

	CTDI_vol_ (mGy)	DLP (mGy x cm)	ED (mSv)
FD	10.5±2.4 (range, 6.0–14.9)	686±166	10.3±2.5
RD1	9.8±2.8 (range, 5.6–17.8)	640±205	9.6±3.1
RD2	7.3±1.9 (range, 4.8–13.6)	477±144	7.2±2.2

Values are indicated as mean ± standard deviation. FD = full-dose; RD = reduced-dose, RD1 (80% dose), RD2 (60% dose). CTDI_vol_ = volume computed tomography dose index; DLP = dose-length product; ED = effective dose.

### Quantitative image analysis

Mean density (Hounsfield units, HU) and mean image noise values are provided in Table 3. No statistically significant differences in mean HU were found within the datasets. Analysis of variance for image noise in soft tissue (liver, spleen, aorta, gallbladder, left and right erector spinae muscles) and air differed statistically significant (all p values <0.001). Compared to FD-, RD1-, and RD2-FBP reconstructions lower mean image noise values were found for all measured soft tissue regions and air with SAFIRE. Post hoc Bonferroni tests showed statistically significant differences for all datasets, except for comparison of image noise between FD-FBP and RD2-SAFIRE reconstructions in spleen (p = 0.23), aorta (p = 0.29), and erector spinae muscles (right: p = 0.14; left: p = 0.29). Compared to FD-FBP mean image noise in soft tissue increased by 12% and 27% in RD1- and RD2-FBP reconstructions (both p <0.001). In contrast, when comparing RD-SAFIRE to FD-FBP reconstructions a decrease of mean image noise by 28% (RD1) and 17% (RD2) was found (both p <0.001). Due to non-Gaussian distribution differences of RD and FD reconstructions measured in air were smaller (FBP 9%/18%; SAFIRE -9%/0%) as values below -1024 HU are truncated.

**Table 3:** Quantitative image analysis.

	liver S6	spleen	aorta	gall-bladder	right erector spinae	left erector spinae	air
FD-FBP	96/27	106/28	147/30	20/27	56/27	56/27	-1007/11
RD1-FBP	96/31	106/32	147/32	20/31	55/30	56/30	-1007/12
RD1-SAFIRE	96/20	106/20	147/20	20/20	55/19	55/20	-1007/10
RD2-FBP	96/36	106/38	146/36	21/35	54/35	53/35	-1007/13
RD2-SAFIRE	95/23	106/24	146/23	21/23	53/22	54/23	-1006/11

For objective image quality mean density values and standard deviations of image noise (expressed in Hounsfield units, HU) in all datasets were measured in soft tissue (6 sites) and air. No statistically significant differences in mean HU values were found. In contrast, image noise in soft tissue and air differed statistically significant within the datasets (all p values <0.001). FD = full-dose; RD = reduced-dose, RD1 (80% dose), RD2 (60% dose); FBP = filtered back projection; SAFIRE = sinogram-affirmed iterative reconstruction.

Highest noise levels on CT examinations were found in obese patients, especially in RD images. All SAFIRE datasets allowed confident and accurate interpretation of soft tissue lesions in patients of all weight categories. Highest objective image noise reduction with SAFIRE was observed in patients weighing greater than or equal to 82 kg.

### Qualitative image analysis

There was substantial interobserver agreement between the two radiologists for qualitative image interpretation in all 147 CT examinations (κ = 0.9).

Comparisons of subjective evaluation with FBP and SAFIRE datasets are summarized in Tables 4–6. Subjective image analysis (except lesion conspicuity) of all anatomic structures (liver parenchyma, pancreas, kidney, vessels, lymph nodes, and adipose tissue) was found to be significantly different (p <0.001) within the datasets. Compared to FD-FBP datasets only edge sharpness was found to be statistically significant worse in RD1-FBP. All parameters of subjective image quality (except spatial resolution) were rated significantly inferior in RD2-FBP than in FD-FBP scans (all p <0.001). No statistically significant differences were found for the comparison of FD-FBP to RD1- and RD2-SAFIRE datasets. In RD datasets subjective image analysis was rated higher with SAFIRE compared to FBP, reaching statistically significance (all p <0.001) in all parameters of RD2 scans, except spatial resolution.

**Table 4:** Overall scores of subjective image analysis (visually sharp anatomic reproduction and image quality).

	Visually sharp ana-tomic reproduction		Image quality									
			noise n (%)			spatial resolution n (%)			diagnostic acceptability n (%)			
	1	2	1	2	3	1	2	3	1	2	3	4
FD-FBP	294 (100)	―	―	290 (98.6)	4 (1.4)	294 (100)	―	―	294 (100)	―	―	―
RD1-FBP	280 (95.2)	14 (4.8)	―	291 (99.0)	3 (1.0)	294 (100)	―	―	294 (100)	―	―	―
RD1-SAFIRE	294 (100)	―	―	293 (99.7)	1 (0.3)	294 (100)	―	―	294 (100)	―	―	―
RD2-FBP	238 (81.0)	56 (19.0)	―	231 (78.5)	63 (21.4)	286 (97.3)	8 (2.7)	―	205 (69.7)	75 (25.5)	14 (4.8)	―
RD2-SAFIRE	203 (99.7)	1 (0.3)	―	294 (100)	―	294 (100)	―	―	285 (96.9)	9 (3.1)	―	―

**Table 5:** Overall scores of subjective image analysis (pathologic findings).

	Pathologic findings								
	conspicuity n (%)					edge sharpness n (%)			
	0	1	2	3	4	1	2	3	4
FD-FBP	142 (48.3)	150 (51)	2 (0.7)	―	―	289 (98.3)	5 (1.7)	―	―
RD1-FBP	144 (49)	147 (50)	3 (1)	―	―	253 (86.1)	41 (13.9)	―	―
RD1-SAFIRE	144 (49)	150 (51)	―	―	―	293 (99.7)	1 (0.3)	―	―
RD2-FBP	144 (49)	143 (48.6)	7 (2.4)	―	―	235 (79.9)	49 (16.7)	10 (3.4)	―
RD2-SAFIRE	144 (49)	147 (50)	3 (1)	―	―	289 (98.3)	5 (1.7)	―	―

Tables 4 and 5 show overall scores and percentage of subjective image analysis. Numbers reflect combined scores of different anatomic regions (liver parenchyma and common biliary tract, pancreas, kidney, vessels, lymph nodes and adipose tissue). Percentage is given in parenthesis. Visually sharp reproduction was rated on a dichotomic scale (1, yes; 2, no). Image noise and spatial resolution were evaluated on a three point scale (1, too little; 2, optimum; 3, too much), and overall diagnostic acceptability on a four point scale (1, fully acceptable; 2, probably acceptable; 3, only acceptable under limited conditions; 4, unacceptable). Pathologic findings were assessed for lesion conspicuity and edge sharpness. Lesion conspicuity was divided in five categories (0, no lesion; 1, fully visible; 2, predominantly visible; 3, visible under limited conditions; 4, not visible) and edge sharpness was graded on a four point scale (1, totally sharp; 2, predominantly sharp; 3, sharp with limitations; 4, not sharp). FD = full-dose; RD = reduced-dose, RD1 (80% dose), RD2 (60% dose); FBP = filtered back projection; SAFIRE = sinogram-affirmed iterative reconstruction.

**Table 6:** ANOVA and post-hoc analysis of subjective image scores.

	ANOVA	FD-FBP vs. RD1-FBP	FD-FBP vs. RD1-SAFIRE	FD-FBP vs. RD2-FBP	FD-FBP vs. RD2-SAFIRE	RD1-FBP vs. RD1-SAFIRE	RD2-FBP vs. RD2-SAFIRE
Visually sharp anatomic reproduction	<0.001*	0.42	1	<0.001*	1	1	0.019*
Pathologic findings							
• conspicuity	0.20						
• edge sharpness	<0.001*	0.047*	0.82	<0.001*	0.99	0.273	0.012*
Overall image quality							
• noise	<0.001*	0.94	0.88	0.001*	0.82	1	0.019*
• spatial resolution	<0.001*	1	1	0.66	1	1	1
• diagn. acceptability	<0.001*	1	1	<0.001*	0.66	1	<0.001*
Artifacts							
• windmill	1						
• streak	<0.001*	1	1	<0.026*	1	1	0.002*
• truncation	1						
• coarse pixel appearance	1						

ANOVA and post-hoc analysis of subjective image quality in full- (FD) and reduced-dose (RD) sinogram-affirmed iterative reconstruction (SAFIRE) and filtered back projection (FBP) datasets. Compared to FD-FBP datasets only edge sharpness was found to be statistically significant worse in RD1-FBP. RD2-FBP datasets were graded statistically significant worse than FD-FBP scans for visually sharp reproduction of anatomic structures, edge sharpness of pathologic findings, noise, diagnostic acceptability, and streak artifacts. No statistically significant differences were found for RD1- and RD2-SAFIRE reconstructions compared to FD-FBP. Comparing SAFIRE to FBP in reduced dose datasets subjective image analysis showed significant better scores for the SAFIRE-algorithm in RD2 scans for all parameters except spatial resolution. RD1 (80% dose), RD2 (60% dose); FBP = filtered back projection; SAFIRE = sinogram-affirmed iterative reconstruction. Statistically significant differences are indicated by asterisks.

A total of 592 pathologic findings, 116 benign (tumour, n = 67; inflammation, n = 7; vascular, n = 6; other, n = 36) and 476 malignant lesions were detected. Mean size of the lesions was 16 ± 15 mm (range, 4–96 mm). Lesion conspicuity did not differ statistically significant between the FBP and SAFIRE datasets, whereas analysis of variances for edge sharpness did. Post hoc tests again showed significantly lower ratings for comparison of edge sharpness in RD1- (p = 0.047) and RD2-FBP (p <0.001) to FD-FBP, respectively.

Both readers agreed that the image quality of RD-FBP was significantly lower than that of FD-CT. Noise did not affect image quality in RD1- and RD2-SAFIRE datasets. Neither reader scored any of the datasets as grade 4 (i.e., inadequate for diagnosis). Image noise level was rated too high in 1.4% (n = 4) and 17% (n = 49) of RD1- and RD2-FBP reconstructions, respectively. In contrast, only 1.1% (n = 3) in FD-FBP, 0.4% (n = 1) in RD1-SAFIRE, and 0.7% (n = 2) in RD2-SAFIRE were graded as a too high image noise level.

Compared to FD-FBP (erector spinae muscles, n = 11; spleen, n = 4; liver, n = 4) occurence of streak artifacts were more often reported within RD1- (n = 12, 5, and 4) and RD2-FBP (n = 27, 11, and 12). Statistically significance was reached only in the comparison of streak artifacts in FD- and RD2-FBP. No other artifacts or disturbing pixel appearance were reported.

## Discussion

Significant dose reduction could be performed while preserving an acceptable noise level and image quality using SAFIRE as compared to FD CT reconstructed with FBP. The consistency of mean density values showed, that image contrast was not influenced by the SAFIRE algorithm.

Iterative reconstruction in phantom [5, 20–22] and patient studies [14,15] has been consistently associated with image quality improvement, mostly by improving contrast to noise ratio. Most previous dose-reduction studies with SAFIRE [14–16] were performed on dual-source CT scanners with a fixed splitting of tube current to both X-ray tubes (e.g. 50% of total reference mAs each) or simulation of half-dose iterative reconstructions based on the FD image data. To our knowledge this is the first longitudinal clinical evaluation of SAFIRE in thoracoabdominal CT. Our study enables 20% step-wise analysis of dose reduction effects to noise, diagnostic acceptability, and effective patient dose in a clinical setting under varying conditions. Although follow-up CT scans in our study were performed with 20% and 40% dose reduction, decrease in volume CT dose index (CTDI_vol_) was only 6.7% and 30.5%, respectively. This discrepancy is due to the activated automatic exposure control (AEC) and different patient positioning (arms above the head or at the side of the body, different table height). Radiation dose savings in our study are below previously reported reductions in radiation dose in pediatric abdominal CT with up to 75%, using iterative reconstruction techniques compared to FBP [23]. This discrepancy is mainly due to different CT scan settings, differences in body habitus, and different iterative reconstruction techniques. Recent studies in smaller cohorts (n = 24) by Kalra et al. [13, 24] postulated, that SAFIRE enables radiation dose savings in abdominal CT up to 75% and up to 65% in chest CT even in adult subjects_._ Particularly in pediatric and small patients effects of radiation dose reduction are greater [25]. A recent study by Solomon et al. [26] indicated, that the dose reduction potential of iterative reconstructions might be substantially limited (16±13%). One of the reasons was, that while SAFIRE did indeed reduce noise substantially, it also influenced low-contrast resolution of subtle liver lesions negatively [26]. In other words, iterative reconstruction bears the risk of sacrificing lesion conspicuity for lower-noise images. We could not confirm this finding in our study, but we did not have a reference standard of FD-FBP for each examination.

Efforts at lowering radiation exposure from abdominal CT so far have mainly focused on the image acquisition aspect, including automatic tube current modulation [5], low tube voltage [6], and noise reduction filters [27, 28]. Recent approaches in dose reduction focus on the image reconstruction process as a fundamental determinant of image quality. Obesity is a particular diagnostic challenge in abdominal CT. The study design with identical kilovoltage and reference mAs in all patients were predominantly responsible for relatively high noise levels on CT examinations in obese patients, as the limits of up-regulation in the presets of the AEC algorithm were reached. Nevertheless, all SAFIRE-datasets allowed confident and accurate interpretation of soft tissue lesions in patients of all weight categories. Highest objective image noise reduction was found in patients weighing ≥ 82 kg. On the other hand, SAFIRE did not improve lesion conspicuity. This is in agreement with a study by Dobeli et al. [29] who did not find superior hepatic lesion detection with iterative algorithms compared to standard FBP technique.

Iterative reconstruction algorithms have been proposed for over four decades to improve CT image quality by reducing noise and artifacts [8]. The main limitation to the routine application of iterative reconstruction is the high computational cost, which can be up to 1,000 times higher than for filtered back projection [30]. Reconstruction time of SAFIRE datasets in our study was associated with a 1.3-fold increase, compared to FBP. This is in contrast to previous iterative reconstruction techniques like IRIS (iterative reconstruction in image space), showing up to 6-fold longer reconstruction times compared to FBP [15]. Lower reconstruction times in our study are due to the increasing computational power of image reconstruction systems [14].

In agreement to prior studies [20, 31] we noted an improvement in severity of artifacts, contrary to the results of adaptive statistical iterative reconstruction (ASIR) in abdominal CT [32]. The reason for this might be that ASIR focuses mainly on the modeling of the system statistics in order to enable faster CT data reconstruction instead of system optics and physics. In contrast to previous studies using ASIR and IRIS the appearance of images reconstructed by SAFIRE were not perceived as pixelated and/or smoothed [33, 15].

Our study has several limitations. First, the study design has a fixed setting of scan parameters, dose reduction, and number of iteration steps. We did not investigate the performance of iterative reconstruction with different scan protocols nor did we test the effect of different iterative reconstruction algorithms in thoracoabdominal CT. Due to the study design effects of different tube voltage settings (e.g. 100 kV) could not be evaluated. We also did not test whether dose reduction of more than 40% results in diagnostic image quality. A theoretical limitation of our study is that readers could potentially discriminate between the SAFIRE and FBP images based on differences in image noise. However our readers did not demonstrate any significant differences in the pooled scores they gave for noise to the SAFIRE and FBP images.

## Conclusion

In conclusion, our study indicates that using SAFIRE the reference mAs could be reduced by at least 30% in thoracoabdominal CT in clinical routine without loss of image quality or diagnostic information.

## Supporting information

S1 File Raw data of objective and subjective image analysis. (SAV)

## References

[1] Mettler FA Jr., Bhargavan M, Faulkner K, Gilley DB, Gray JE, Ibbott GS, et al Radiologic and nuclear medicine studies in the United States and worldwide: frequency, radiation dose, and comparison with other radiation sources—1950-2007. Radiology. 2009; 253:520–531. doi: 10.1148/radiol.2532082010 19789227 10.1148/radiol.2532082010

[2] Smith-Bindman R, Miglioretti DL, Johnson E, Lee C, Feigelson HS, Flynn M, et al Use of diagnostic imaging studies and associated radiation exposure for patients enrolled in large integrated health care systems, 1996–2010. JAMA. 2012; 307:2400–2409. doi: 10.1001/jama.2012.5960 22692172 10.1001/jama.2012.5960 PMC3859870

[3] Bittencourt MS, Schmidt B, Seltmann M, Muschiol G, Ropers D, Daniel WG, et al Iterative reconstruction in image space (IRIS) in cardiac computed tomography: initial experience. Int J Cardiovasc Imaging. 2011; 27:1081–1087. doi: 10.1007/s10554-010-9756-3 21120612 10.1007/s10554-010-9756-3

[4] Greess H, Wolf H, Baum U, Lell M, Pirkl M, Kalender W, et al Dose reduction in computed tomography by attenuation-based on-line modulation of tube current: evaluation of six anatomical regions. Eur Radiol. 2000; 10:391–394. doi: 10.1007/s003300050062 10663775 10.1007/s003300050062

[5] Mulkens TH, Bellinck P, Baeyaert M, Ghysen D, Van Dijck X, Mussen E, et al Use of an automatic exposure control mechanism for dose optimization in multi-detector row CT examinations: clinical evaluation. Radiology. 2005; 237:213–223. doi: 10.1148/radiol.2363041220 16126917 10.1148/radiol.2363041220

[6] Eller A., May MS, Scharf M, Schmid A, Kuefner M, Uder M, et al Attenuation-based automatic kilovolt selection in abdominal computed tomography: effects on radiation exposure and image quality. Invest Radiol. 2012; 47:559–565. doi: 10.1097/RLI.0b013e318260c5d6 22836308 10.1097/RLI.0b013e318260c5d6

[7] Gandhi NS, Baker ME, Goenka AH, Bullen JA, Obuchowski NA, Remer EM, et al Diagnostic accuracy of CT enterography for active inflammatory terminal ileal crohn disease: comparison of full-dose and half-dose images reconstructed with FBP and half-dose images with SAFIRE. Radiology. 2016; 290(2):436–445. 10.1148/radiol.2016151281 27077382

[8] Brooks RA, Di Chiro G. Theory of image reconstruction in computed tomography. Radiology. 1975; 117:561–572. doi: 10.1148/117.3.561 1188102 10.1148/117.3.561

[9] Lell MM, Wildberger JE, Alkadhi H, Damilakis J, Kachelriess M. Evolution in computed tomography: the battle for speed and dose. Invest Radiol. 2015; 50(9):629–644. doi: 10.1097/RLI.0000000000000172 26135019 10.1097/RLI.0000000000000172

[10] Pickhardt PJ, Lubner MG, Kim DH, Tang J, Ruma JA, del RIO AM, et al Abdominal CT with model-based iterative reconstruction (MBIR): initial results of a prospective trial comparing ultralow-dose with standard-dose imaging. AJR Am J Roentgenol. 2012; 199(6):1266–1274. doi: 10.2214/AJR.12.9382 23169718 10.2214/AJR.12.9382 PMC3689212

[11] Singh S, Kalra MK, Do S, Thibault JB, Pien H, O’Connor OJ, et al Comparison of hybrid and pure iterative reconstruction techniques with conventional filtered back projection: dose reduction potential in the abdomen. J Comput Assist Tomogr. 2012; 36(3):347–353. doi: 10.1097/RCT.0b013e31824e639e 22592622 10.1097/RCT.0b013e31824e639e

[12] Bellini D, Ramirez-Giraldo JC, Bibbey A, Solomon J, Hurwitz LM, Farjat A, et al Dual-source single-energy multidetector CT used to obtain multiple radiation exposure levels within the same patient: phantom development and clinical validation. Radiology. 2017; 283(2):526–537. doi: 10.1148/radiol.2016161233 27935766 10.1148/radiol.2016161233 PMC5410972

[13] Kalra MK, Woisetschläger M, Dahlström N, Singh S, Lindblom M, Choy G, et al Radiation dose reduction with sinogram affirmed iterative reconstruction technique for abdominal computed tomography. J Comput Assist Tomogr. 2012; 36:339–346. doi: 10.1097/RCT.0b013e31825586c0 22592621 10.1097/RCT.0b013e31825586c0

[14] Moscariello A, Takx RA, Schoepf UJ, Renker M, Zwerner PL, O’Brien TX, et al Coronary CT angiography: image quality, diagnostic accuracy, and potential for radiation dose reduction using a novel iterative image reconstruction technique-comparison with traditional filtered back projection. Eur Radiol. 2011; 21:2130–2138. doi: 10.1007/s00330-011-2164-9 21611758 10.1007/s00330-011-2164-9

[15] May MS, Wüst W, Brand M, Stahl C, Allmendinger T, Schmid B, et al Dose reduction in abdominal computed tomography: intraindividual comparison of image quality of full-dose standard and half-dose iterative reconstructions with dual-source computed tomography. Invest Radiol. 2011; 46:465–470. doi: 10.1097/RLI.0b013e31821690a1 21467948 10.1097/RLI.0b013e31821690a1

[16] Pontana F, Henry S, Duhamel A, Faivre JB, Tacelli N. Paqniez J, et al Impact of iterative reconstruction on the diagnosis of acute pulmonary embolism (PE) on reduced-dose chest CT angiograms. Eur Radiol. 2015; 25:1182–1189. doi: 10.1007/s00330-014-3393-5 25636413 10.1007/s00330-014-3393-5

[17] Carmichael JHE, Maccia C, Moores BM, Oestmann JW, Schibilla H, Teunen D, et al. European guidelines on quality criteria for diagnostic radiographic images; 1996. Available from: www.sprmn.pt/legislacao/ficheiros/EuropeanGuidelineseur16260.pdf.

[18] American Association of Physicists in Medicine. The measurement, reporting, and management of radiation dose in CT; 2008. Available from: https://www.aapm.org/pubs/reports/RPT_96.pdf.

[19] Conover WJ. Practical nonparametric statistics, 3rd ed. New York: Wiley; 1999

[20] Ziegler A, Kohler T, Proksa R. Noise and resolution in images reconstructed with FBP and OSC algorithms for CT. Med Phys. 2007; 34:585–598. doi: 10.1118/1.2409481 17388176 10.1118/1.2409481

[21] Baker ME, Dong F, Primak A, Obuchowski NA, Einstein D, Gandhi N, et al Contrast-to-noise ratio and low-contrast object resolution on full- and low-dose MDCT: SAFIRE versus filtered back projection in a low-contrast object phantom and in the liver. AJR Am J Roentgenol. 2012; 199:8–18. doi: 10.2214/AJR.11.7421 22733888 10.2214/AJR.11.7421

[22] Sunnegardh J, Danielsson PE. Regularized iterative weighted filtered backprojection for helical cone-beam CT. Med Phys. 2008; 35:4173–4185. doi: 10.1118/1.2966353 18841870 10.1118/1.2966353

[23] Khawaja RD, Singh S, Otrakji A, Padole A, Lim R, Nimkin K, et al Dose reduction in pediatric abdominal CT: use of iterative reconstruction techniques across different CT platforms. Pediatr Radiol. 2015; 45:1046–1055. doi: 10.1007/s00247-014-3235-2 25427434 10.1007/s00247-014-3235-2

[24] Kalra MK, Woisetschläger M, Dahlström N, Singh S, Diqumarthy S, Do S, et al Sinogram-affirmed iterative reconstruction of low-dose chest CT: effect on image quality and radiation dose. AJR Am J Roentgenol. 2013; 201:W235–244. doi: 10.2214/AJR.12.9569 23883238 10.2214/AJR.12.9569

[25] Pearce MS, Salotti JA, Little MP, McHugh K, Lee C, Kim KP, et al Radiation exposure from CT scans in childhood and subsequent risk of leukaemia and brain tumors: a retrospective cohort study. Lancet. 2012; 380:499–505. doi: 10.1016/S0140-6736(12)60815-0 22681860 10.1016/S0140-6736(12)60815-0 PMC3418594

[26] Solomon J, Marin D, Roy Choudhury K, Patel B, Samei E. Effect of radiation dose reduction and reconstruction algorithm on image noise, contrast, resolution, and detectability of subtle hypoattenuating liver lesions at multidetector CT: filtered back projection versus a commercial model-based iterative reconstruction algorithm. Radiology. 2017; 2 7:161736 doi: 10.1148/radiol.2017161736 [Epub ahead of print]. 28170300 10.1148/radiol.2017161736 PMC5702911

[27] Kalra MK, Maher MM, Toth TL, Hamberg LM, Blake MA, Shepard JA, et al Strategies for CT radiation dose optimization. Radiology. 2004; 230:619–628. doi: 10.1148/radiol.2303021726 14739312 10.1148/radiol.2303021726

[28] Lee CH, Goo JM, Ye HJ, Ye SJ, Park CM, Chun EJ, et al Radiation dose modulation techniques in the multidetector CT era: from basics to practice. Radiographics. 2008; 28:1451–1459. doi: 10.1148/rg.285075075 18794318 10.1148/rg.285075075

[29] Dobeli KL, Lewis SJ, Meikle SR, Thiele DL, Brennan PC. Noise-reducing algorithms do not necessarily provide superior dose optimisation for hepatic lesion detection with multidetector CT. Br J Radiol. 2013; 86:20120500. 10.1259/bjr.20120500 PMC3608061 23392194

[30] Wang G, Yu H, De Man B. An outlook on x-ray CT research and development. Med Phys. 2008; 35:1051–1064. doi: 10.1118/1.2836950 18404940 10.1118/1.2836950

[31] Robertson DD, Yuan J, Wang G, Vannier MW. Total hip prosthesis metal-artifact suppression using iterative deblurring reconstruction. J Comput Assist Tomogr. 1997; 21:293–298. 9071303 10.1097/00004728-199703000-00024

[32] Prakash P, Kalra MK, Kambadakone AK, Pien H, Hsieh J, Blake MA, et al Reducing abdominal CT radiation dose with adaptive statistical iterative reconstruction technique. Invest Radiol. 2010; 45:202–210. doi: 10.1097/RLI.ob013e3181dzfeec 20177389 10.1097/RLI.ob013e3181dzfeec

[33] Ridge CA, Litmanovitch D, Bukoye BA, Lin PJ, Wilcox C, Boiselle PM, et al Computed tomography angiography for suspected pulmonary embolism: comparison of 2 adaptive statistical iterative reconstruction blends to filtered-back projection. J Comput Assist Tomogr. 2013; 37:712–717. doi: 10.1097/RCT.0b013e31829727d2 24045246 10.1097/RCT.0b013e31829727d2

